# Mitigation of lampricide toxicity to juvenile lake sturgeon: the importance of water alkalinity and life stage

**DOI:** 10.1093/conphys/coz089

**Published:** 2019-12-08

**Authors:** Scott L J Hepditch, Laura R Tessier, Jonathan M Wilson, Oana Birceanu, Lisa M O’Connor, Michael P Wilkie

**Affiliations:** 1 Department of Biology and Institute for Water Science, Wilfrid Laurier University, 75 University Avenue West, Waterloo, ON N2L 3C5, Canada; 2 Great Lakes Laboratory for Fisheries and Aquatic Sciences, Fisheries and Oceans Canada, 1219 Queen Street East, Sault Ste. Marie, ON P6A 2E5, Canada

**Keywords:** Invasive species, 3-trifluoromethyl-4-nitrophenol, Sea lamprey control, Acipenser fulvescens, Allometry, Gill

## Abstract

The pesticide, 3-trifluoromethyl-4-nitrophenol (TFM), is used to control invasive sea lamprey (*Petromyzon marinus*) populations in the Laurentian Great Lakes. Applied to infested tributaries, it is most toxic to larval sea lamprey, which have a low capacity to detoxify TFM. However, TFM can be toxic to lake sturgeon (*Acipenser fulvescens*), whose populations are at risk throughout the basin. They are most vulnerable to TFM in early life stages, with the greatest risk of non-target mortality occurring in waters with high alkalinity. We quantified TFM toxicity and used radio-labelled TFM (^14^C-TFM) to measure TFM uptake rates in lake sturgeon in waters of different pH and alkalinity. Regardless of pH or alkalinity, TFM uptake was 2–3-fold higher in young-of-the-year (YOY) than in age 1-year-plus (1+) sturgeon, likely due to higher mass-specific metabolic rates in the smaller YOY fish. As expected, TFM uptake was highest at lower (pH 6.5) versus higher (pH 9.0) pH, indicating that it is taken up across the gills by diffusion in its unionized form. Uptake decreased as alkalinity increased from low (~50 mg L^−1^ as CaCO_3_) to moderate alkalinity (~150 mg L^−1^ as CaCO_3_), before plateauing at high alkalinity (~250 mg L^−1^ as CaCO_3_). Toxicity curves revealed that the 12-h LC_50_ and 12-h LC_99.9_ of TFM to lake sturgeon were in fact higher (less toxic) than in sea lamprey, regardless of alkalinity. However, in actual treatments, 1.3–1.5 times the minimum lethal TFM concentration (MLC = LC_99.9_) to lamprey is applied to maximize mortality, disproportionately amplifying TFM toxicity to sturgeon at higher alkalinities. We conclude that limiting TFM treatments to late summer/early fall in waters of moderate-high alkalinity, when lake sturgeon are larger with lower rates of TFM uptake, would mitigate non-target TFM effects and help conserve populations of these ancient, culturally important fishes.

## Teaser

Lay Summary

Young-of-the-year lake sturgeon living in waters of high alkalinity are susceptible to mortality when exposed to 3-trifluoromethyl-4-nitrophenol (TFM), a pesticide used to control invasive sea lamprey in the Great Lakes. This risk may be reduced, however, by delaying TFM treatments to late-summer/fall, when sturgeon are larger and accumulate TFM more slowly.

## Introduction

The lake sturgeon (*Acipenser fulvescens*) is endemic to the Central United States, the Great Lakes-St. Lawrence region and the Hudson Bay drainages of Canada (Harkness and Dymond, 1961; Scott and Crossman, 1973; Peterson *et al.*, 2007). Once abundant within their natural ranges, populations were decimated by the early 1900s due to overfishing and by dams and navigation locks built in the 1800s and early 1900s (Harkness and Dymond, 1961; Auer, 1999; Peterson *et al.*, 2007). Few healthy populations of lake sturgeon currently exist in North America, due in part to the time required for sexual maturation (~12–15 and 8–27 years in males and females, respectively), the associated low rates of recruitment and continued habitat degradation (Thuemler, 1988; Peterson *et al.*, 2007; Pollock *et al.*, 2015). Conservation and restoration efforts have been hampered by ongoing anthropogenic disturbances including the destruction of historic spawning grounds and critical downstream habitats (Auer, 1996; Wilson and McKinley, 2004). Nevertheless, lake sturgeon restoration efforts are underway in parts of the Great Lakes-St. Lawrence, with particular success in the Lake Huron-Lake Erie Corridor (Pollock *et al.*, 2015; Welsh *et al.*, 2017).

Lake sturgeon population recovery efforts could be complicated by sea lamprey (*Petromyzon marinus*) control efforts in the Great Lakes (O’Connor *et al.*, 2017; Dobiesz *et al.*, 2018). Sea lampreys are jawless fish that spend most of their life as relatively sedentary, filter-feeding larvae (ammocoetes) burrowed in the substrate of streams. After 3–7 years, they undergo a profound metamorphosis characterized by the formation of a multi-toothed oral disc and rasping tongue which are used by the juvenile sea lampreys to attach to large-bodied fishes and feed on their blood as parasites, or predators when they kill their hosts (Beamish and Potter, 1975; Youson, 2003; Renaud et al., 2009). Native to the North Atlantic Ocean, sea lampreys likely became established in Lake Ontario in the 1800s, although whether or not this was a naturally occurring or invasive population is a subject of considerable debate (see Eshenroder 2014 for review). It is widely accepted, however, that modifications and improvements to the Welland Canal allowed sea lampreys to bypass Niagara Falls and gain entry from Lake Ontario into Lake Erie and the upper Great Lakes in the early 20th century (Lawrie, 1970; Eshenroder, 2009). The subsequent explosion of invasive sea lampreys led to massive losses of culturally and economically important fisheries in all of the Great Lakes, most notably the populations of lake trout (*Salvelinus namaycush*), which had virtually collapsed by the mid-1950s (Lawrie, 1970; Siefkes, 2017).

In response to the sea lamprey invasion, Canada and the USA created the Great Lakes Fishery Commission (GLFC) in 1955, which was given the responsibility to come up with means to eradicate or control sea lamprey populations (Crowe, 1975, Fetterolf, 1980; GLFC, 2011). Control measures that were implemented have included in-stream barriers, sterile male release and chemical control using lampricides (McLaughlin *et al.*, 2007; Siefkes, 2017; Wilkie *et al.*, 2019). Today, the lampricide 3-trifluoromethyl-4-nitrophenol (TFM) constitutes the backbone of the sea lamprey control program (McDonald and Kolar, 2007; Siefkes, 2017; Wilkie *et al.*, 2019). It is applied to tributaries infested with larval sea lampreys, which have a lower capacity to detoxify TFM compared to most non-target fishes (Lech and Statham, 1975; Kane *et al.*, 1994; Bussy *et al.*, 2018a,b).

Lake sturgeon, however, are particularly vulnerable to TFM in their early life stages (Boogaard *et al.*, 2003). They have been identified in 57 different rivers subjected to sea lamprey control operations, with historical evidence of lake sturgeon presence in 40 additional tributaries (O’Connor *et al.*, 2017). Because they occupy the same reaches of streams as larval sea lamprey or ammocoetes, which live burrowed in the stream substrate as filter feeders, juvenile lake sturgeon can be exposed to potentially lethal amounts of TFM. Indeed, the 12-h LC_50_ of juvenile lake sturgeon less than 100 mm in length overlaps with that of larval sea lamprey (McDonald and Kolar, 2007).

**Figure 1:**
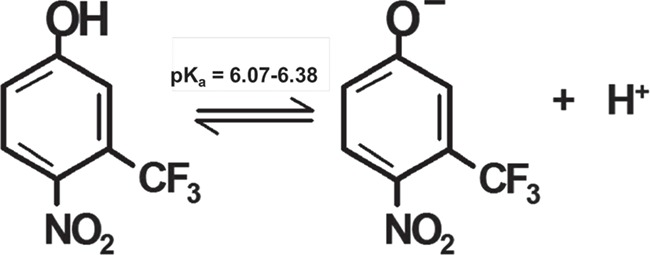
Structure and dissociation equilibrium of TFM (pK_a_ = 6.07–6.38). TFM is an ionizable weak acid, in which the proportion of the unionized (phenolic) moiety increases as water pH decreases. As water pH increases above the pK_a_, the proportion of the ionized (phenolate) form predominates

Another complication is that the tolerance of lake sturgeon to TFM appears to decrease relative to sea lamprey in waters of high alkalinity, in which much higher concentrations of TFM are needed to maximize sea lamprey eradication (Boogaard *et al.*, 2003; O’Connor *et al.*, 2017; Dobiesz *et al.*, 2018). Alkalinity refers to the ability of water to buffer changes in pH due to the presence of HCO_3_^−^, CO_3_^2−^ or other ions (Cole, 1988) and should not be confused with the term ‘alkaline’ which is used to describe solutions of basic pH (pH > 7.0). In sea lampreys and other fishes including brown trout (*Salmo trutta*), tolerance to TFM increases with alkalinity but the underlying mechanisms are not well understood. This is in contrast to water pH, in which TFM toxicity increases with decreases in water pH (Bills *et al.*, 2003). As a weak acid, with a pKa of 6.07 to 6.38 (Hubert, 2003; McConville *et al.*, 2016), the proportion of unionized TFM compared to ionized TFM increases as water pH decreases (Fig. 1), resulting in greater diffusive uptake of TFM across the gills of rainbow trout (*Oncorhynchus mykiss*) and sea lamprey in more acidic water (Hunn and Allen, 1974; Hlina *et al.*, 2017). Presumably, lake sturgeon respond to changes in water pH in a similar manner to other fishes, but this has not been tested. Nor have any studies been able to demonstrate why TFM toxicity decreases with increasing alkalinity in lamprey, let alone other non-target species such as lake sturgeon. This study proposes to better understand how water chemistry affects TFM uptake by lake sturgeon and could provide us with additional insight into why they are more vulnerable to TFM at higher alkalinity compared to other fishes. The objectives of our study were to (i) determine how alkalinity and pH influenced sturgeon tolerance to TFM and (ii) explain any observed differences in TFM tolerance in waters of different alkalinity by measuring rates of TFM uptake and accumulation by juvenile lake sturgeon.

The lake sturgeon’s greater TFM sensitivity in early compared to later life stages is also unexplained. One possibility is that due to their small size, fingerling lake sturgeon would have greater mass-specific metabolic rates resulting in greater uptake of TFM (Goolish, 1991; Killen *et al.*, 2010). Tessier *et al.* (2018) demonstrated that rates of TFM uptake per unit body mass by larval sea lamprey scaled inversely with body size, but this has not been examined in other fishes. Thus, a final objective was to test the hypothesis that smaller young-of-the-year (YOY) lake sturgeon would have higher rates of TFM accumulation, making them more vulnerable to TFM toxicity than those older than 1-year (1+) animals.

## Method and materials

### Experimental animals and holding

Lake sturgeon (*Acipenser fulvescens*) eggs and milt were collected from wild fish in the spring of 2016 and 2017 and the eggs fertilized at the Sustainable Sturgeon Culture, Emo, ON (Courtesy of Joe Hunter, Rainy River First Nation), and transported by plane to Pearson International Airport, Toronto, ON, and then to the Alma Research Station (ARS; University of Guelph, Alma, ON, Canada). The eyed eggs were hatched and reared using facilities at the ARS following the Genoa National Fish Hatchery Lake Sturgeon Culture Standard Operating Procedures manual (Aloisi et al. 2006). Prior to experiments, sturgeon of the appropriate age and size [YOY < 100 mm; 1+ fish >130 mm) were transported from the ARS to Wilfrid Laurier University (WLU), Waterloo, ON. They were housed under a 12-h light: 12-h dark cycle for a minimum of 3 weeks in a G-Hab fish holding system (Pentair Aquatic Eco-Systems, Apopka, FL, USA; total volume ~ 600 L), filled with recirculating dechlorinated City of Waterloo tap water (pH ~ 8.0; titratable alkalinity ~ 250 mg L^−1^ as CaCO_3_; hardness ~ 350 mg L^−1^ as CaCO_3_; temperature ~ 14–16°C). The water replacement rate was 10% of the system volume per day, and the GHAB was equipped with temperature control, biological and mechanical filtration and UV sterilization.

The lake sturgeon were fed daily with a slurry containing bloodworms (larval chironomids; San Francisco Bay Brand, Inc., Newark, CA, USA) and EWOS starter feed (Cargill Incorporated, Minneapolis, MN, USA) at 2% body mass per day for each, but were fasted overnight prior to experiments to minimize fouling of the water due to defecation and to reduce the accumulation of excreted ammonia. All experiments and fish husbandry followed the Canadian Council of Animal Care guidelines and were approved by the Wilfrid Laurier University Animal Care Committee. The movement of lake sturgeon within the Province of Ontario was reviewed and approved by the Ontario Introductions and Transfers Committee.

### Experimental protocols

#### Effects of water alkalinity on lake sturgeon survival during TFM exposure

One-year-plus (1+) old lake sturgeon (*N* = 63; length = 138.8 ± 1.6 mm, mass = 7.8 ± 1.9 g) were acclimated to nominal water alkalinities of 50 mg L^−1^ as CaCO_3_ (low alkalinity), 100 mg L^−1^ as CaCO_3_ (moderate) or 250 mg L^−1^ as CaCO_3_ (high) for 1 week and subjected to an acute survival test at a nominal TFM concentration of 3.5 mg L^−1^. This value was within the range of concentrations likely to result in different survival rates and was within the range likely to be encountered by lake sturgeon during typical lampricide treatments (Bills *et al.*, 2003; O’Connor *et al.*, 2017). Water of the appropriate chemistry was prepared by mixing dechlorinated municipal tap water with reverse osmosis water at the Cold Regions and Water Science Centre at WLU followed by measurements of water pH, alkalinity, dissolved O_2_ and temperature.

The survival test setup comprised 37-L glass aquaria filled with water of the appropriate alkalinity, which were placed in a water bath to maintain temperature at 15°C. The night before experiments, each aquarium, containing no fish, was dosed with TFM (measured concentration = 3.61 ± 0.2 mg L^−1^), at which time concentrations were measured, to verify that the amounts of TFM were within 10% of the target concentration, and again the next morning to ensure that concentrations were stable (Table 1). The lake sturgeon were then transferred in groups of five to aquaria of the appropriate alkalinity in triplicate (*N* = 15 at low, moderate and high alkalinity, respectively) and survival monitored continuously for the first 8 h of the experiment and then hourly for the next 8 h and at 24 h. Simultaneous control fish at each alkalinity (*N* = 6 at low, moderate or high alkalinity, respectively) were also monitored in the absence of TFM. Dead fish were immediately removed from the TFM exposure tanks, and the time of death was recorded, followed by measurements of the mass and length of each animal and collection of water samples for determination of water chemistry and TFM concentration. Surviving animals were euthanized after 24 h with a lethal dose of tricaine methanesulfonate (TMS; 1.5 g L^−1^; Syndel Labs, Nanaimo, BC, Canada) buffered in 3.0 g L^−1^ NaHCO_3_, and the carcasses disposed of as per institutional policies and procedures.

**Table 1:** Water alkalinity, pH, temperature measurements and measured TFM concentrations used to test the effects of water alkalinity on 1+ lake sturgeon survival during exposure to a nominal TFM concentration of 3.5 mg L^−1^

**Alkalinity**				
**Nominal** (mg L^−1^ as CaCO_3_)	**Measured** (mg L^−1^ as CaCO_3_)	**pH**	**Temperature** (°C)	**[TFM]** (mg L^−1^)
**Low alkalinity**				
50 mg L^−1^ as CaCO_3_—control	51.0 ± 0.0	8.03 ± 0.03	15.1 ± 0.2	—
50 mg L^−1^ as CaCO_3_ plus TFM	52.2 ± 0.4	7.92 ± 0.05	14.6 ± 0.2	3.62 ± 0.08
***Moderate alkalinity***				
100 mg L^−1^ as CaCO_3_—control	97.5 ± 8.1	8.03 ± 0.03	15.1 ± 0.2	—
100 mg L^−1^ as CaCO_3_ plus TFM	109.1 ± 2.5	8.05 ± 0.03	14.8 ± 0.2	3.57 ± 0.03
***High alkalinity***				
250 mg L^−1^ as CaCO_3_—control	233.8 ± 4.3	8.16 ± 0.16	15.2 ± 0.1	—
250 mg L^−1^ as CaCO_3_ plus TFM	229.5 ± 3.3	8.23 ± 0.04	15.3 ± 0.1	3.57 ± 0.09

Data presented as the mean ± standard error of the mean (SEM). *N* = 4 measurements for controls, and *n* = 8–12 for experimental treatments (exposed to TFM).

#### Effects of pH and alkalinity on TFM uptake

Two groups of lake sturgeon, YOY (*N* = 150; length = 76 ±1 mm; mass = 1.4 ± 0.1 g) and 1+ (*N* = 150; length = 136 ±1 mm; mass = 8.5 ± 0.01 g), were acclimated for 1 week to a set alkalinity and pH (see Table 2 for acclimation conditions). The fish were acclimated in 37-L glass aquaria (*N* = 25 per aquaria), which continuously received reconstituted water of the appropriate chemistry and temperature that continuously emptied into each aquarium at a rate of 0.1–0.2 L min^−1^ from a 400-L head tank. The artificial water was produced daily in 400-L batches following American Society of Testing Materials (2000) methods. Identical amounts of CaSO_4_, KCl and MgSO_4_ were added to reverse osmosis water to maintain the same water hardness (101.1 ± 2.6 mg L^−1^ as CaCO_3_) and Ca^2+^, K^+^ and Cl^−^ concentrations across all experiments, and NaHCO_3_ was added to achieve desired alkalinities. Diluted HCl (0.5–2.0 N) and NaOH (0.5–2.0 N) were added to establish the desired pH.

**Table 2:** Summary of water chemistry measurements taken for separate groups of young-of-the-year (YOY) and age 1+ lake sturgeon acclimated to different water alkalinities and pHs for 1 week

**Age**	**Experimental treatment**	**Alkalinity** (mg L^−1^ as CaCO_3_)	**pH**	**[Ca** ^**2+**^ **]** (mg L^−1^)	**Hardness** (mg L^−1^ as CaCO_3_)	**Temperature** (°C)
YOY	Low alkalinity	57 ± 1	8.00 ± 0.01	17	98	14.2 ± 0.2
YOY	Medium alkalinity	152 ± 1	8.18 ± 0.02	16	98	14.3 ± 0.2
YOY	High alkalinity	251 ± 1	8.37 ± 0.01	14	96	14.2 ± 0.1
YOY	Low pH	145 ± 2	6.57 ± 0.06	17	110	14.1 ± 0.3
YOY	Medium pH	152 ± 1	8.18 ± 0.02	16	98	14.3 ± 0.2
YOY	High pH	152 ± 1	8.95 ± 0.04	17	111	14.8 ± 0.2
1+	Low alkalinity	51 ± 1	8.00 ± 0.01	16	114	14.8 ± 0.2
1+	Medium alkalinity	152 ± 1	8.23 ± 0.03	17	95	14.7 ± 0.2
1+	High alkalinity	250 ± 1	8.40 ± 0.01	14	94	14.2 ± 0.3
1+	Low pH	150 ± 1	6.51 ± 0.02	17	90	14.9 ± 0.3
1+	Medium pH	152 ± 1	8.23 ± 0.03	17	95	14.7 ± 0.2
1+	High pH	151 ± 1	8.91 ± 0.06	16	105	14.8 ± 0.1

Data (where applicable) presented as the mean ± standard error of the mean (SEM).

Rates of TFM uptake were measured using ^14^C-TFM, based on established methods (Blewett *et al.*, 2013; Tessier *et al.*, 2018). One day prior to each set of experiments, lake sturgeon (*N* = 10) were transferred to individual darkened, rectangular flux chambers (dimensions = 6 × 10 × 14 cm; volume = 750 mL; *N* = 1 lake sturgeon per container) contained in an identical system to that described above. Each flux chamber continuously received water of the appropriate alkalinity and pH (Table S1—Supplementary data) at a flow rate ~0.1 L min^−1^ and were left overnight to acclimate to their surroundings. Next morning, water flow was cut off to each container, and the volume adjusted to exactly 750 mL. Appropriate amounts of ^14^C-TFM (provided courtesy of T. Hubert, Upper Midwest Environmental Sciences Center, US Geological Survey, La Crosse, WI, USA; DuPont/New England Nuclear, DE, USA) and non-radioactive TFM (TFM; 35% active ingredient in isopropanol; provided courtesy of the Sea Lamprey Control Centre (SLCC), Department of Fisheries and Oceans Canada; Clariant SFC GmbH Werk, Griesheim, Germany) were then added to establish nominal TFM concentrations of 1.0, 2.5, 5.0 or 10 mg L^−1^, with mean specific activity (MSA) of 110.8 ± 0.3 counts per minute (CPM) nmol^−1^ TFM. These nominal concentrations best represented the range of TFM concentrations used to treat lamprey-infested streams in the Great Lakes at specific water pHs and alkalinities (Bills *et al.*, 2003; McDonald and Kolar, 2007).

Three different concentrations of TFM (low, medium and high) were used to determine if TFM uptake was dose-dependent under the different water chemistry conditions used. After a 15-min mixing period, water samples were collected at 0 and 1 h for determination of the non-radioactive TFM concentration and ^14^C-TFM radioactivity. Preliminary experiments had shown that a 1-h exposure minimized TFM-induced mortality at higher TFM concentrations and that this was the optimum exposure time for sufficient ^14^C-TFM to accumulate in the fish. Immediately after, the fish were euthanized with an overdose of 1.5 g L^−1^ of TMS buffered in 3.0 g L^−1^ NaHCO_3_ and then washed in concentrated, non-radioactive TFM (10 mg L^−1^) to remove residual radioactive (‘hot’) TFM from the body surface, followed by a rinse with deionized water. Measurements of body mass and the standard length were collected before transfer to individual 50-mL polypropylene centrifuge tubes (Conical Centrifuge Tubes, Corning Falcon™, NY, USA), followed by subsequent processing for whole body radioactivity measurements.

Despite the relatively short ^14^C-TFM exposure period, we were concerned that simultaneous clearance of ^14^C-TFM-labelled TFM or its metabolites by the fish could result in an underestimation of TFM uptake rates. Accordingly, a subset of lake sturgeon were treated with salicylamide, a known inhibitor of the process of glucuronidation, thought to be a primary method of TFM detoxification in non-target fishes (Lech and Statham, 1975; Kane *et al.*, 1994) and lake sturgeon (Bussy *et al.*, 2018a). Lake sturgeon (*N* = 10; length = 96.3 ± 0.8 mm; mass = 3.1 ± 0.1 g) were first acclimated for 1 week to moderate pH (8.14 ± 0.03) and alkalinity (150 ± 1 mg L^−1^ as CaCO_3_) and then exposed to 25 mg L^−1^ salicylamide for 2 h immediately prior to measuring TFM uptake using the methods described above (Lech, 1974). TFM uptake was also measured in a group of parallel control fish (*N* = 10) acclimated and exposed to TFM under the same conditions, without salicylamide. It was predicted that if TFM uptake were underestimated due to excretion of (biotransformed) ^14^C-TFM via the gastrointestinal tract or renal routes, then TFM uptake rates would be higher following salicylamide treatment. Passive losses of TFM across the gills would not be expected, as there would be a large inwardly directed TFM gradient during the first hour of TFM exposure.

### Analytical methods

#### Water chemistry and TFM concentration

Water alkalinity and pH were measured using commercial kits (Hach, Alkalinity Test Kit, Model AL-AP, Hach Canada, Mississauga, ON) and a handheld pH meter (pH 11 meter, Oakton Instruments, IL, USA). Water Ca^2+^ and Mg^2+^ concentrations were measured using flame atomic absorption spectroscopy (AAS, PinAAcle 900T, Perkin Elmer, Waltham, MA, USA). Water TFM concentration was measured at a wavelength of 395 nm using a Novaspec II spectrophotometer (Pharmacia Biotech, Cambridge, UK), following the Standard Operating Procedures of the Sea Lamprey Control Centre (IOP: 012.4), Fisheries and Oceans Canada, Sault Ste. Marie, ON.

#### Whole body beta radiation measurements

Following experiments, whole, intact lake sturgeon were digested in 10 times their body mass of 1 mol L^−1^ HNO_3_ for 2 days at 60°C. The digested samples were intermittently vortexed during the digestion process, followed by centrifugation for 5 min at ~1200 × *g*. A sub-sample of the resulting supernatant (2 mL) was added to 4 mL of Ultima Gold ™ AB organic scintillation cocktail (PerkinElmer, MA, USA) in duplicate and left overnight in the dark to minimize chemiluminescence prior to measuring the beta radioactivity (LC6500 Multi-Purpose Scintillation Counter, Beckman Coulter, CA, USA). Rates of uptake were calculated according to Tessier *et al.* (2018) using the following equation:

(1) \begin{equation*}\textrm{TFM Uptake Rate} = \textrm{CPM}_{\textrm{sturgeon}}/(\textrm{MSA} \times \Delta\textrm{T})\end{equation*}

where uptake is measured in nmol g^−1^ h^−1^, CPM is the total whole-body radioactivity in CPM of ^14^C-TFM g^−1^ body mass, MSA is the specific activity of the water samples (CPM nmol^−1^ of TFM) and Δ*T* is the time of exposure (h).

### Statistical analyses

Survival curves, ± 95% confidence intervals (CIs), were generated using survival data collected over 24-h exposure of 1+ lake sturgeon to TFM at different water alkalinities. Log-rank (Mantel–Cox) tests were used to determine if the family of resulting TFM survival curves were significantly different from one another at the *P* < 0.05 level. Pairwise comparisons between different curves were then made using a Bonferroni-corrected threshold value of *P* < 0.0083, determined by dividing the overall level of significance (*P* < 0.05) by the total number of comparisons (*K* = 6). To compare differences in the rates of mortality in the different treatment groups, a hazard-risk ratio (relative slope of the survival curves) was calculated using the Mantel–Haenszel method.

TFM uptake data were presented as the mean ± 1 SEM against the mean TFM concentration ± 1 SEM. One-way ANOVA was used to determine if there were significant differences among mean rates of TFM uptake with increasing TFM concentration at the different water pHs or alkalinities tested. When significant variation was observed among the mean uptake rates, pair-wise comparisons were made using a Holm–Sidak post-test (*P* < 0.05). In instances where there were significant differences in the standard deviation of the sample set, non-parametric ANOVA (Welch’s ANOVA) followed by a Dunnet’s post-test was used (*P* < 0.05).

Because TFM exposure concentrations varied between experiments, direct statistical comparisons between TFM uptake rates at different water chemistries (pH or alkalinity) were not possible. Instead, scatter plots were used to depict the relationships between water chemistry and rates of TFM uptake with increases in water TFM concentration for individual YOY or 1+ lake sturgeon, followed by linear regression analysis. The slopes of the TFM uptake *vs* TFM concentration relationships were considered significantly different from one another if the corresponding confidence intervals did not overlap. All statistical analysis was conducted using Prism 8.0 (GraphPad Inc., San Diego, CA, USA).

## Results

### Effects of alkalinity on TFM tolerance in lake sturgeon

**Figure 2:**
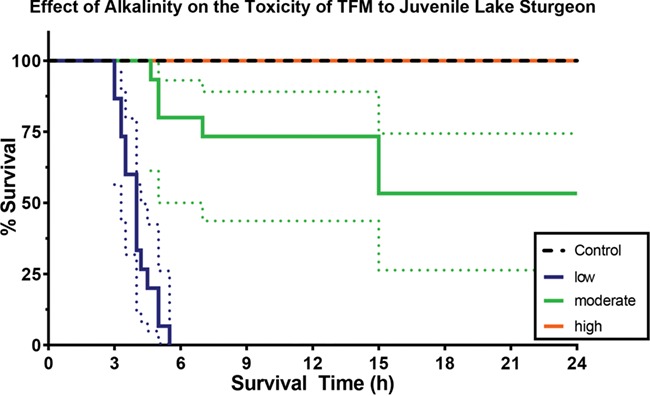
Effects of water alkalinity on survival of lake sturgeon exposed to TFM. Percent survival of 1-year-plus (1+) old lake sturgeon exposed to a nominal TFM concentration of 3.5 mg L^−**1**^ for 24 h (measured overall mean [TFM] = 3.61 ± 0.2) in waters of low (blue line; 50 mg L^−**1**^ as CaCO_3_; *N* = 15), moderate (green line; 100 mg L^−**1**^ as CaCO_3_; *N* = 15) or high (orange line; 250 mg L^−**1**^ as CaCO_3_; *N* = 15) alkalinity. No mortalities were observed in control fish at any alkalinity (dashed line; *N* = 6 per alkalinity). Dotted lines represent the 95% confidence interval

Exposure of age 1+ lake sturgeon to TFM (measured overall mean concentration = 3.61 ± 0.02 mg L^−1^) in waters of low alkalinity (52.2 ± 0.4 mg L^−1^ as CaCO_3_), moderate alkalinity (109.1 ± 2.5 mg L^−1^ as CaCO_3_) or high alkalinity (229.5 ± 3.3 mg L^−1^ as CaCO_3_) generated significantly different survival profiles (Fig. 2; *P* < 0.0001). At low alkalinity, all fish died between 3 and 6 h of TFM exposure. Fish exposed to TFM at moderate alkalinity experienced only partial mortality, which occurred at a slower pace. Survival in these fish approximated 75% between 4.5 and 7.0 h of the exposure, before dropping to slightly greater than 50% after 15 h, with no further mortality thereafter (Fig. 2). Pairwise comparisons of low versus moderate alkalinity survival curves were significantly different from one another (*P* < 0.0001; level of significance set to *P* = 0.0083). Survival was 100% in lake sturgeon exposed to TFM at high alkalinity and under control (non-exposed) conditions at all three alkalinities (Fig. 2). Calculations of the hazard ratio indicated that the expected rate of mortality in fish exposed to TFM in low alkalinity was 47 times greater than those at high alkalinity and 17 times greater than those at moderate alkalinity (data not shown). Further details on the water chemistry and TFM exposure concentrations are presented in Table 1.

### Effects of pH on rates of TFM uptake by YOY and 1+ lake sturgeon

Prior exposure of lake sturgeon to salicylamide, to inhibit glucuronidation of TFM (Lech, 1974), had no significant effect on rates of TFM uptake (*P* = 0.707). The uptake rate of 11.8 ± 0.4 nmol g^−1^ h^−1^ observed in the lake sturgeon exposed to salicylamide was similar to the rate of 10.9 ± 0.6 nmol g^−1^ h^−1^ measured in the non-treated fish (data not shown).

**Figure 3:**
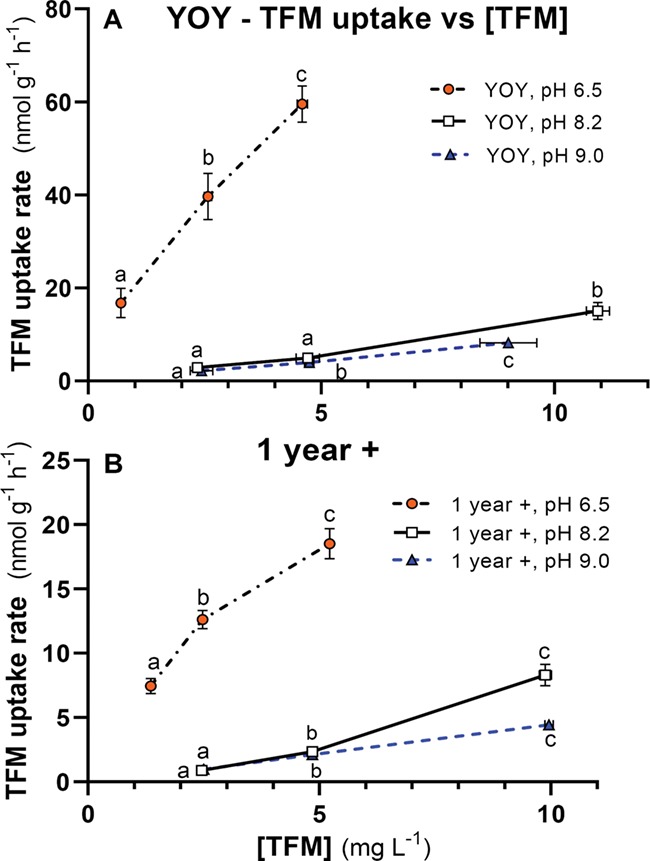
Changes in TFM uptake with TFM concentration at different water pHs in young-of-the-year (YOY) and 1-year-old (1+) lake sturgeon. Rates of TFM uptake for (**A**) YOY lake sturgeon (~9 months) and (**B**) age 1+ lake sturgeon (~15 months) at nominal water pHs of 6.5 (orange, filled circles), 8.2 (open squares) and 9.0 (blue triangles) vs TFM concentration. Measured TFM concentrations and TFM uptake rates are reported as the mean ± 1 SEM (*N* = 8–10 per treatment). In cases where SEMs are very low, error bars are obscured by symbols and not visible. Different letters denote statistically significant differences in TFM uptake vs TFM concentration at a given pH (one-way ANOVA, Holm–Sidak or Dunn’s multiple comparisons tests as appropriate, *P* < 0.05)

Water pH had a pronounced effect on rates of TFM uptake *versus* TFM concentration in both YOY and 1+ lake sturgeon. In YOY lake sturgeon acclimated to low pH (measured pH = 6.50 ± 0.02), TFM uptake increased more than 2-fold from a rate of 16.8 ± 3.2 nmol g^−1^ h^−1^ at a nominal TFM concentration of 0.5 mg L^−1^ to 39.6 ± 5.0 nmol g^−1^ h^−1^ (*P* = 0.003) at a nominal TFM concentration of 2.5 mg L^−1^, and a further 50% to 59.5 ± 3.9 nmol g^−1^ h^−1^ at the highest concentration of 5 mg L^−1^ (Fig. 3A; *P* < 0.0001). The rate of TFM uptake increase with TFM concentration was much lower at moderate water pH (measured pH = 8.19 ± 0.01) and higher pH (measured pH = 9.03 ± 0.01) than at lower pH. At moderate pH, TFM uptake averaged 2.9 ± 0.4 nmol g^−1^ h^−1^ at the lowest nominal TFM concentration of 2.5 mg L^−1^, increasing to 15.0 ± 1.8 nmol g^−1^ h^−1^ at a nominal TFM concentration of 10 mg L^−1^ (Fig. 3A; *P* < 0.0001). At high pH, TFM uptake averaged 2.2 ± 0.1 nmol g^−1^ h^−1^ at a nominal TFM concentration of 2.5 mg L^−1^, steadily increasing to a rate of 9.0 ± 0.6 at the highest nominal TFM concentration of 10 mg L^−1^ (Fig. 3A; *P* < 0.0001).

Similar trends were observed in 1+ lake sturgeon, in which TFM uptake rates were greatest in the fish acclimated to low pH (measured pH = 6.53 ± 0.01), in which the greatest rate of uptake averaged 18.5 ± 1.1 nmol g^−1^ h^−1^ at a nominal TFM concentration of 5 mg L^−1^ (Fig. 3B). Rates were much lower in the fish acclimated to moderate pH (measured pH = 8.19 ± 0.01) and high pH (measured pH = 8.97 ± 0.01), averaging 8.3 ± 0.8 and 4.4 ± 0.3 nmol g^−1^ h^−1^, respectively, at nominal TFM concentrations of 10 mg L^−1^ (Fig. 3B). TFM uptake was also dose-dependent in this group, significantly increasing with TFM concentration at all three pHs tested (Fig. 3B). Compared to the smaller, YOY lake sturgeon, TFM uptake rates measured at moderate and high pH were 50–75% lower in the larger, 1+ animals (compare Fig. 3A and B keeping in mind differences in axis scales).

**Figure 4:**
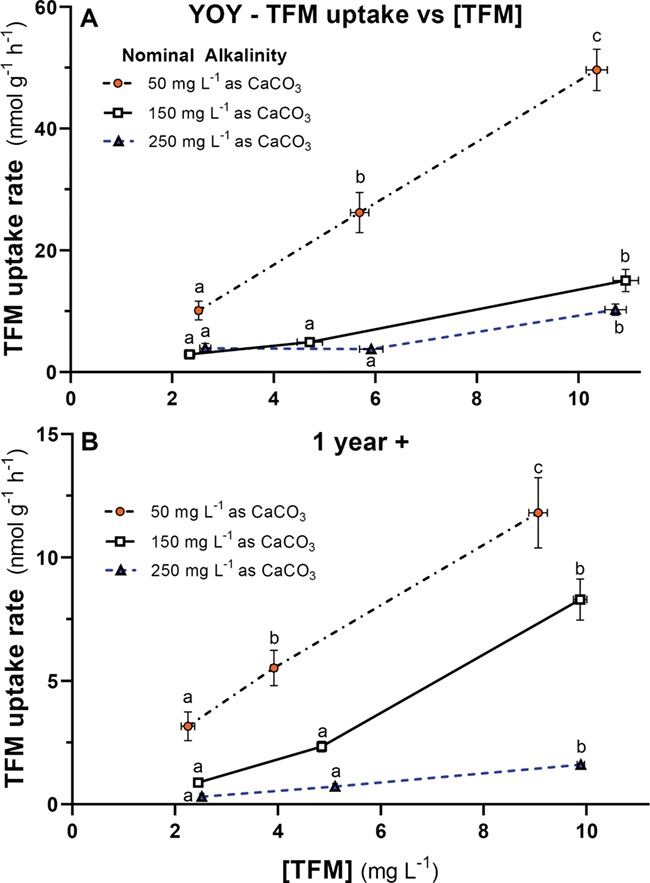
Changes in TFM uptake with TFM concentration at different water alkalinities in young-of-the-year (YOY) and 1-year-old (1+) lake sturgeon. Rates of TFM uptake for (**A**) YOY lake sturgeon (~9 months) and (**B**) age 1+ lake sturgeon (~15 months) at nominal water alkalinities of 50 (orange, filled circles), 150 (open squares) or 250 (blue triangles) mg L^−**1**^ as CaCO_3_ vs TFM concentration. Measured TFM concentrations and TFM uptake rates are reported as the mean ± 1 SEM (*N* = 8–10 per treatment). In cases where SEMs are very low, error bars are obscured by symbols and not visible. Different letters denote statistically significant differences in TFM uptake vs TFM concentration at a given alkalinity (one-way ANOVA, Holm–Sidak multiple comparisons test; *P* < 0.05)

### Effects of alkalinity on the rates of TFM uptake by YOY and 1+ lake sturgeon

The rate of TFM uptake was markedly influenced by alkalinity and more so in the YOY than in the 1+ lake sturgeon. In the YOY lake sturgeon, a clear dose-dependent response was observed in the fish acclimated to low alkalinity, where TFM uptake significantly increased with each step-up in TFM concentration (Fig. 4A). At low alkalinity (57 ± 1 mg L^−1^ as CaCO_3_), rates of TFM uptake in the YOY fish averaged 10.1 ± 1.6 nmol g^−1^ h^−1^ at a nominal TFM concentration of 2.5 mg L^−1^ and increased approximately 2.5-fold to 26.2 ± 3.3 nmol g^−1^ h^−1^ at a TFM concentration of 5.0 mg L^−1^ (*P* = 0.0003) and a further 2-fold to 49.7 ± 3.4 nmol g^−1^ h^−1^ at the highest TFM concentration of 50 mg L^−1^ (Fig. 4A; *P* < 0.0001). At moderate alkalinity (152 ± 1 mg L^−1^ as CaCO_3_), the changes in rates of TFM uptake with increasing TFM concentration were less pronounced. At the lowest nominal concentration of TFM (2.5 nmol g^−1^ h^−1^), TFM uptake averaged 2.9 ± 0.4 nmol g^−1^ h^−1^ and was not significantly different from the rate of 4.9 ± 0.4 nmol g^−1^ h^−1^ measured at a nominal TFM concentration of 5 mg L^−1^ (*P* = 0.210). However, TFM was significantly elevated at the highest TFM exposure concentration of 10 mg L^−1^, averaging 15.0 ± 1.8 nmol g^−1^ h^−1^ (Fig. 4A; *P* < 0.0001). At the highest alkalinity (251 ± 1 mg L^−1^ as CaCO_3_), rates of TFM uptake did not significantly change as the nominal concentrations of TFM were increased from 2.5 to 5.0 mg L^−1^, averaging 3.9 ± 0.8 and 3.8 ± 0.4, respectively. However, TFM uptake was significantly elevated when the TFM concentration was increased further to a nominal concentration of 10 mg L^−1^, when rates averaged 10.3 ± 0.9 nmol g^−1^ h^−1^ (Fig. 4A; *P* < 0.0001).

As with the YOY lake sturgeon, TFM uptake was greatest in the 1+ lake sturgeon exposed to TFM at low alkalinity (measured alkalinity = 52 ± 1 mg L^−1^ as CaCO_3_). In both low and moderate alkalinity (152 ± 1 mg L^−1^ as CaCO_3_), rates of TFM uptake significantly increased with increasing TFM concentration, peaking at 11.8 ± 1.4 and 8.3 ± 0.8 nmol g^−1^ h^−1^, respectively, at the highest nominal TFM concentration 10 mg L^−1^ (*P* < 0.0001; Fig. 4B). While the rates of uptake were also dose-dependent in waters of high alkalinity (measured alkalinity = 252 ± 1 mg L^−1^ as CaCO_3_), uptake rates averaged only 1.6 ± 0.1 nmol g^−1^ h^−1^ at a TFM concentration of 10 mg L^−1^, which were just above the levels of detection, as compared to the low and moderate alkalinity treatment groups (Fig. 4B). At all three alkalinities, the rates of TFM uptake were 40–80% lower in the larger 1+ than smaller YOY lake sturgeon at comparable TFM concentrations (Compare Fig. 4A to 4B).

## Discussion

### The effects of alkalinity on TFM toxicity

**Figure 5:**
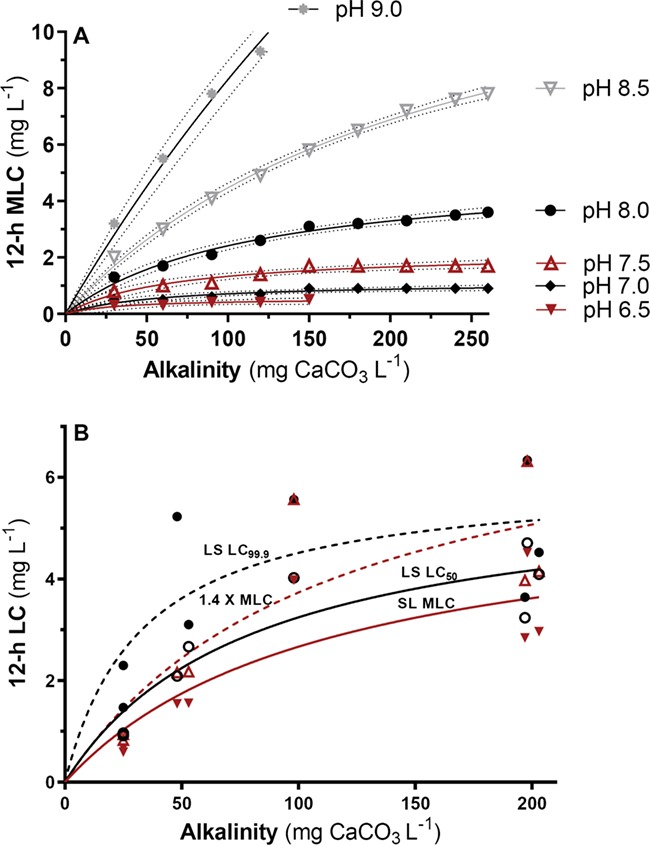
Effects of water alkalinity on TFM toxicity. (**A**) Data re-plotted from Bills *et al.* (2003) relating water alkalinity to the 12-h minimum lethal concentration of TFM (MLC = 12-h LC_99.9_) in larval sea lamprey at different water pHs. Data were fitted using non-linear regression (GraphPad Prism, ver. 8.0). (**B**) Relationship between water alkalinity and the 12-h MLC (red solid line; solid triangles) and 1.4 times the 12-h MLC (red dashed line; open triangles) of sea lamprey (SL), and the 12-h LC_50_ (solid black line; open circles) and LC_99.9_ (dashed black line; solid circles) of lake sturgeon (LS). Data replotted from O’Connor *et al.* (2017)

The present study demonstrates that lake sturgeon are more tolerant to TFM when exposed to the lampricide in waters of high compared to low alkalinity due to reductions in the rate of TFM uptake. Higher alkalinity therefore reduces the toxicity of TFM to lake sturgeon, as it does in sea lamprey and non-target fishes such as brown trout (*S. trutta*; Bills *et al.*, 2003). The relationship between water pH and uptake was also consistent with observations made in lamprey and rainbow trout, increasing with decreases in pH at a given TFM concentration because of a higher proportion of the unionized, more diffusible form of TFM in more acidic water (discussed further below; Hunn and Allen, 1974; Bills *et al.*, 2003; Hlina *et al.*, 2017). The relationship between alkalinity and TFM toxicity is more complicated, however, partly because alkalinity generally increases with water pH. However, when the effects of alkalinity on TFM toxicity are corrected for pH in the laboratory, the relationship between larval sea lamprey TFM tolerance and alkalinity is hyperbolic (Fig. 5A; Bills *et al.*, 2003). At relatively low alkalinities, less than 100 mg L^−1^ as CaCO_3_, the MLC of sea lamprey rises rapidly with alkalinity before plateauing as water alkalinity approaches 200–250 mg L^−1^ as CaCO_3_, which is near the upper limit for Great Lakes tributaries containing lake sturgeon (O’Connor *et al.*, 2017).

Similar analyses of toxicity and water quality data collected by O’Connor *et al.* (2017) in the field shows similar trends for both sea lamprey and YOY lake sturgeon. Due to the nature of field experiments, the data were more variable than the laboratory data presented here. However, it was clear that the MLC (12-h LC_99.9_) of sea lamprey and the corresponding 12-h LC_50_ and 12-h LC_99.9_ of YOY lake sturgeon in the field also increased in a curvilinear fashion with increases in alkalinity, plateauing at alkalinities greater than 200 mg L^−1^ as CaCO_3_ (Fig. 5B). Notably, the 12-h LC_50_ and LC_99.9_ of the YOY lake sturgeon was greater than the MLC of the sea lamprey at all alkalinities. At first glance, these findings appear to contradict those of O’Connor *et al.* (2017), who reported that as alkalinity increased, the probability of lake sturgeon survival decreased when exposed to 1.4 times the MLC of sea lamprey, a typical dose used for sea lamprey control. However, at 1.4 times the MLC, the toxicity curve for sea lamprey shifted up and away from the lake sturgeon 12-h LC_50_ curve, approaching and eventually intersecting with the LC_99.9_ of YOY lake sturgeon at alkalinities between 150 and 200 mg L^−1^ as CaCO_3_. At lower alkalinities, this 1.4-factor has less effect because the absolute increases in TFM dose are less than those required at higher alkalinities, making lake sturgeon more vulnerable to toxicity at those concentrations (Fig. 5B).

### Effects of water chemistry on TFM uptake by juvenile lake sturgeon

**Figure 6:**
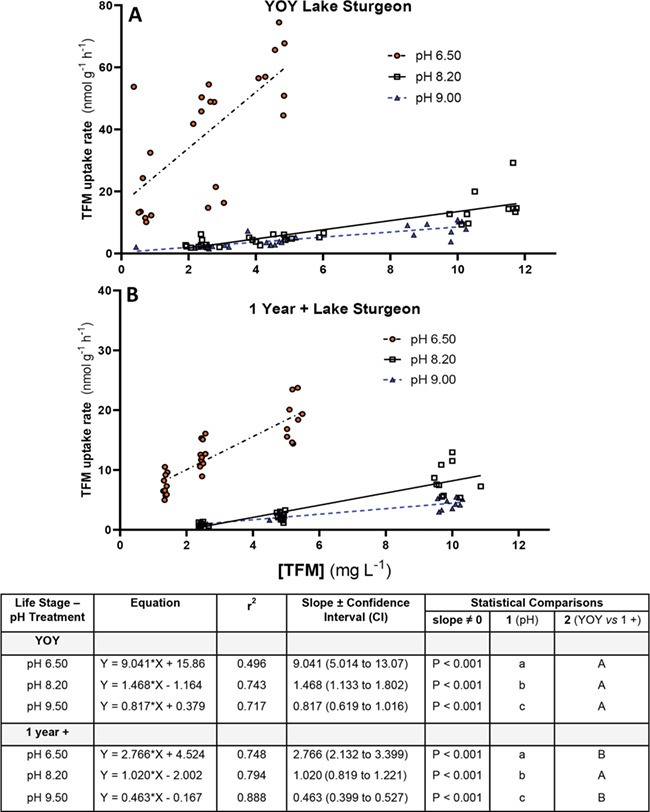
Linear regression relationships between TFM uptake and TFM concentration at different water pHs and life stages in lake sturgeon. Using the data depicted in Fig. 3, the TFM uptake rate vs TFM concentration relationships for individual (**A**) YOY and (**B**) age 1+ lake sturgeon were plotted at nominal water pHs of 6.50 (orange, filled circles), 8.20 (open squares) and 9.00 (blue triangles). Lines of best of fit were determined using linear regression, with the corresponding equations, *r*^2^ and slope (plus confidence interval) displayed in the box below. The slopes of all relationships were significantly different from zero (*N* = 28–30 at each water pH per life stage). Different lowercase letters (Comparison 1) denote statistically significant differences in the slope of the TFM uptake vs concentration relationship at a given pH; different uppercase letters (Comparison 2) represent significant differences between the slope of the TFM uptake vs concentration relationship between YOY and age 1+ lake sturgeon at a given water pH (*P* < 0.05)

As expected, TFM uptake was inversely related to water pH in both YOY and 1+ lake sturgeon due to pH-dependent effects on the speciation of TFM. This is further illustrated by the linear regression analysis depicted in Fig. 6, in which the rates of TFM uptake of each fish are plotted against TFM concentration at each acclimation pH. The most striking observations were the markedly higher rates of TFM uptake in the low pH acclimated *vs* moderate and high pH acclimated fish. In addition, the 6- to 11-fold greater slope of the relationship at low compared to moderate and high pH also indicates that TFM uptake is much more sensitive to even small deviations in TFM concentrations under such conditions. This implies that even slight variations in the concentration of TFM in lower pH waters could result in much greater rates of TFM uptake by lake sturgeon, and greater sensitivity to toxicity.

With a pK_a_ of between 6.07 and 6.38 (Hubert, 2003; McConville *et al.*, 2016), the amount of TFM in its unionized (phenolic) form and its ionized (phenolate ion) form changes with water pH (Hunn and Allen, 1974; McDonald and Kolar, 2007; Hlina *et al.*, 2017). As a weak acid, a greater proportion of TFM is in its unionized, more lipophilic form at lower pHs than at higher pHs (Fig. 1). Thus, the significantly greater rates of TFM uptake for juvenile lake sturgeon exposed to TFM in water of lower pH (6.5) compared to higher pH (9.0) support the original hypothesis that TFM is primarily taken up in its unionized (phenolic) form across the gills (Hunn and Allen, 1974; Hlina *et al.*, 2017). This finding is also consistent with well-established observations that the toxicity of TFM is greater in lamprey and non-target fishes at lower pH (Marking and Olson, 1975; Bills *et al.*, 2003), as well as in lake sturgeon (O’Connor *et al.*, 2017).

**Figure 7:**
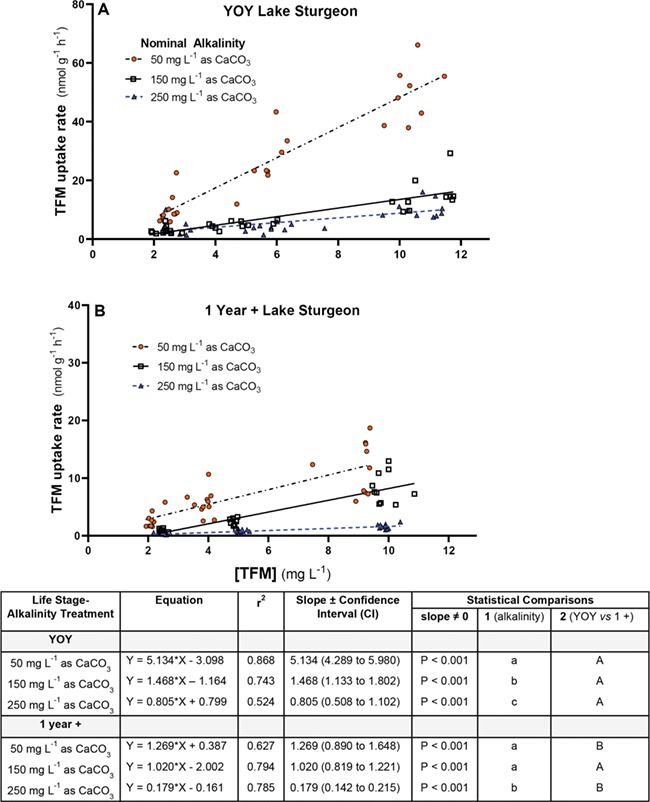
Linear regression relationships between TFM uptake and TFM concentration at different water alkalinities and life stages in lake sturgeon. Using the data depicted in Figure 4, the TFM uptake rate vs TFM concentration relationships for individual (**A**) YOY and (**B**) age 1+ lake sturgeon were plotted at nominal water alkalinities of 6.50 (orange, filled circles), 8.20 (open squares) and 9.00 (blue triangles). Lines of best of fit were determined using linear regression, with the corresponding equations, *r*^2^ and slope (plus confidence interval) displayed in the box below. The slopes of all relationships were significantly different from zero (*N* = 28–30 at each water alkalinity per life stage). Different lowercase letters (Comparison 1) denote statistically significant differences in the slope of the TFM uptake vs concentration relationship at a given alkalinity; different uppercase letters (Comparison 2) represent significant differences between the slope of the TFM uptake vs concentration relationship between YOY and age 1+ lake sturgeon at a given water a given water alkalinity (*P* < 0.05)

The present study demonstrates that at a given concentration of TFM, increasing alkalinity results in a decrease in TFM uptake. This is demonstrated by the linear regression analysis depicted in Fig. 7, in which the slopes of the TFM uptake *vs* TFM concentration relationship were significantly greater at low compared to high alkalinity for both YOY and age 1+ lake sturgeon. By definition, alkalinity is a measure of water buffer capacity (Cole, 1988), which means that the addition of acidic equivalents to water will have less effect on water pH as alkalinity increases. As inspired water is pumped across the gills of fishes, the pH is altered by the hydration of CO_2_ to H^+^ and HCO_3_^−^ and by the excretion of metabolic acid (H+) or base (HCO_3_^−^, NH_3_) equivalents (Wright *et al.*, 1986; Playle and Wood, 1989; Lin and Randall, 1990). In lower-alkalinity water, H^+^ and CO_2_ excretion would lead to greater acidification of the expired water, resulting in lower water pH in the gill microenvironment, higher concentrations of unionized TFM and therefore greater TFM uptake. In contrast, the excretion CO_2_ of acidic equivalents by the gills will have less effect on the pH of the gill microenvironment as alkalinity (buffer capacity) increases. Thus, the pH-dependent changes in TFM speciation in the gill microenvironment will be reduced at higher alkalinities, resulting in less unionized TFM and lower uptake, as was observed in the present study. This was the situation reported for rainbow trout exposed to chlorinated phenols, in which differences between the pH of inspired and expired water at higher alkalinity were lower leading to lower rates of chlorinated phenol uptake due to reductions in the proportion of unionized phenols in the gill water (Erickson *et al*., 2006a,b). Future experiments aimed at quantifying how water pH in the gill microenvironment is influenced by water alkalinity could prove to be very informative for predicting how the speciation and bioavailability of TFM, and other ionizable xenobiotics, influence toxicity in fishes.

### Effects of age and body size on rates of TFM uptake and sensitivity

The reductions in TFM uptake that were observed as pH was increased from pH 6.5 to pH 9.0 were greater than 80% in both YOY and 1+ animals, which was unsurprising given the strong link between TFM speciation and pH described above. However, the situation was markedly different for alkalinity, which had much more pronounced effects on the rates of TFM uptake by YOY lake sturgeon than in the much larger 1+ animals. In the YOY animals, an increase in alkalinity from ~50 to 150 mg CaCO_3_ L^−1^ resulted in marked reductions in the mean rates of TFM uptake, with absolute decreases ranging from ~32–40 nmol g^−1^ h^−1^ at the highest TFM concentration (nominal = 10 mg L^−1^), whereas the absolute decreases in TFM uptake were only 1/10 to one quarter of these values in the 1+ animals. That the step from moderate to high alkalinity water only reduced TFM uptake slightly suggests that any additional interactions affecting TFM uptake and likely TFM toxicity would be limited above 150 mg CaCO_3_ L^−1^.

A surprising observation was the very large differences in the rates of TFM uptake by YOY compared to 1+ lake sturgeon. In cases where both groups were exposed to similar TFM concentrations, the respective mean rates of TFM uptake by YOY lake sturgeon were two to four times greater than the 1+ animals acclimated to identical water pHs, and three to six times greater in YOY sturgeon acclimated to identical alkalinities. The corresponding linear regression analyses, in which the slopes of the TFM uptake *vs* TFM concentration relationship were approximately 2–4-fold greater in the YOY compared to the age 1+ fish at different water pHs and alkalinities, also demonstrated that the greater TFM sensitivity of YOY lake sturgeon compared to larger, 1+ lake sturgeon is related to their much higher rates of TFM uptake (Figs 6 and 7). The finding is probably a consequence of the much smaller masses of the YOY lake sturgeon (mean = 1.4 g; range = 0.5–3.4 g) compared to their larger, 1+ counterparts (mean = 8.5 g; range = 5.2–16.2 g).

**Figure 8:**
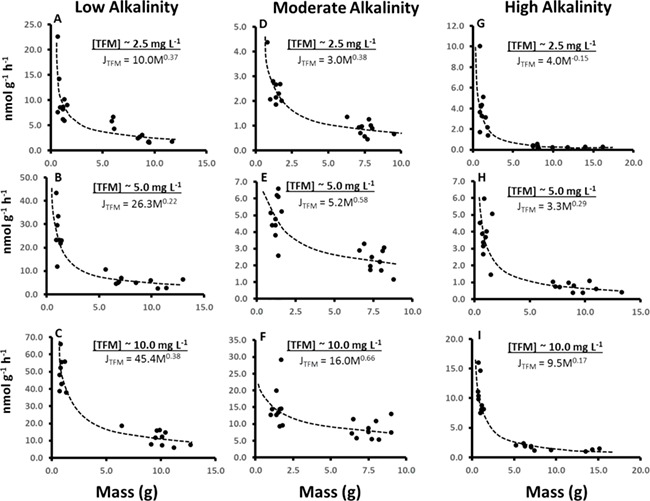
Effects of body size on TFM uptake by juvenile lake sturgeon. Scatter gram depicting rates of TFM uptake vs body mass for individual YOY and 1+ lake sturgeon exposed to nominal TFM concentrations of 2.5, 5.0 and 10 mg L^−1^ at low (nominal = 50 mg L^−1^ as CaCO3), moderate (nominal = 150 mg L^−1^ as CaCO_3_) or high alkalinity (nominal = 250 mg L^−1^ as CaCO_3_). Data fitted using Power relationship (Microsoft Excel). Refer to discussion for details of calculations

To examine this relationship further, we plotted the relationship between mass-specific TFM uptake and body mass separately at the different alkalinities we tested, which revealed that the mass-specific rates of TFM uptake decreased exponentially as body mass increased (Fig. 8). Allometric power relationships were then determined by plotting log TFM uptake versus log body mass (not shown) to better describe how TFM uptake was affected by body mass. It is well established that metabolic rate is an allometric or power function of body mass, described by the equation *Y* = *a*M*^b^*, where *Y* is the dependent variable such as *Ṁ*O_2_, or in this case TFM uptake, and *M* is animal mass, with *b* being a mass (aka. scaling) exponent which corrects for changes in Y with body mass. In the case of metabolic rate_,_ the proportionately constant, *a*, is derived from log–log plots of whole body oxygen consumption rate (*Ṁ*O_2_) versus *M* (Kleiber, 1947; Peters, 1983; Glazier, 2013). Unlike proportional relationships, where the mass exponent, *b*, is near 1, for ṀO_2_*b* usually has a value close to 0.75 in mammals, invertebrates and fishes (Hill and Potter, 1970; Goolish, 1991; Wilkie *et al.*, 2001; Nelson, 2016). However, this was not the case with TFM uptake as *b* was much lower than 0.75 in all cases, falling between 0.22 and 0.38 in low alkalinity water (Fig. 8A–C), 0.38 to 0.66 at moderate alkalinity (Fig. 8D–F), and − 0.15 and 0.29 at high alkalinity (Fig. 8G–I). Similar low values for *b* were observed in larval sea lamprey exposed to TFM, in which it was suggested that TFM uptake is disproportionately greater in smaller larval sea lamprey (Tessier *et al.*, 2018), which also appears to be the case in YOY lake sturgeon.

The higher rates of TFM uptake by smaller compared to larger lake sturgeon was likely related to their greater relative oxygen demands. Meta-analysis of several species of sturgeon indicated that mass-specific *Ṁ*O_2_ decreased exponentially with body mass in sturgeon, as in other fish species (Peake 2005). Thus, we hypothesize that the higher rates of TFM uptake in the smaller YOY lake sturgeon were likely a result of the correspondingly higher rates of gill ventilation relative to their larger 1+ counterparts. Tessier *et al.* (2018) also recently demonstrated that TFM uptake by larval sea lamprey was tightly correlated with routine rates of oxygen consumption, which is likely the case in lake sturgeon but requires further investigation. It should be noted that in studies focusing on YOY lake sturgeon and green sturgeon (*Acipenser medirostris*), *b* fell between 0.9 and 1.0 suggesting that rates of oxygen consumption may not follow the typical allometric relationship as tightly in their early life stages as in other fishes (Allen and Cech, 2007; Svendsen *et al.*, 2014). Nevertheless, a priority of future research should be to learn more about the respiratory physiology of these ancient fishes for not only its basic value, but because of the insight that may be gained about how these fishes are affected by other xenobiotics, in addition to lampricides.

The present findings suggest that the greater sensitivity of YOY compared to 1+ lake sturgeon to TFM is related to greater rates of TFM uptake by the smaller, younger fish, but this may not be the only contributing factor. Although lake sturgeon are known to clear TFM following glucuronidation (LeClair, 2014), salicylamide-exposed lake sturgeon did not have rates of TFM uptake that were significantly different than controls, suggesting that clearance via glucuronidation did not influence the rates of uptake measured during the TFM exposures. However, the greater sensitivity of YOY lake sturgeon to TFM could also be related to a lower, relative capacity to detoxify TFM. Lake sturgeon have been shown to detoxify TFM using Phase II biotransformation, which includes the conjugation of TFM to TFM-glucuronide using glucuronidation (LeClair, 2014; Bussy *et al.*, 2018a), as observed in channel catfish (*Ictalurus punctatus*), bluegill (*Lepomis macrochirus*) and rainbow trout (Lech, 1974; Lech and Statham, 1975; Marking and Olson, 1975; Kane *et al.*, 1994; Bussy *et al.* 2018a,b). However, the capacity of lake sturgeon to utilize uridine diphosphate glucuronyltransferase (UDPGT) as compared to TFM tolerant species, such as the rainbow trout or bluegill (Kane *et al.*, 1994), is not known. Studies aimed at understanding more about the ability of lake sturgeon to use Phase II, not to mention Phase I, pathways of biotransformation could shed further light on how lake sturgeon cope with TFM, not to mention other xenobiotics, could be very important for lake sturgeon conservation measures.

### Implications for lake sturgeon conservation and sea lamprey control

The present findings could have important implications for reducing or eliminating the possible influence of sea lamprey control on lake sturgeon conservation and restoration efforts in the Great Lakes. These efforts are complicated by the lake sturgeon’s late sexual maturation (18–27 years in females) and infrequent spawning, which occurs every 4–9 years in females (Harkness and Dymond, 1961; Fortin *et al.*, 1993; Peterson *et al.*, 2007). One option, to reduce the concentration of TFM during applications, was implemented by the US Fish and Wildlife Service in the early 2000s. This policy required sea lamprey control crews to use lower concentrations of TFM, the MLC rather than 1.3–1.5 times the MLC, to treat lamprey-infested streams containing lake sturgeon (Adair and Sullivan, 2009). While this lowered rates of lake sturgeon mortality, there was concern that there were corresponding increases in the numbers of residual sea lamprey that survived treatment. Another issue was the possibility of increased predation by sea lamprey on lake sturgeon, which could offset any reductions in lampricide-induced mortality (Dobiesz et al. 2018). Further, laboratory studies had shown that attacks on lake sturgeon reduced growth and condition factor, as well as causing lethality (Patrick *et al.*, 2009; Sepulveda *et al.*, 2012). As a result of these concerns, the ‘sturgeon protocol’ for TFM treatment was stopped and sea lamprey TFM treatment concentrations returned to 1.3–1.5 times the MLC in rivers containing lake sturgeon, while still retaining treatment of these rivers to August 1 or later, when lake sturgeon are expected to be greater than >100 mm in length (O’Connor et al. 2017).

The present findings, however, lend strong support to another management option that has been implemented and could be expanded to conserve vulnerable lake sturgeon populations, without compromising sea lamprey control efforts. The exponential declines we observed for TFM uptake with body size strongly suggest that delaying treatment of high alkalinity sea lamprey/lake sturgeon streams until the late summer or early autumn, when YOY lake sturgeon are much larger and could lower adverse effects and non-mortality in these fishes. Another advantage to this approach is the greater protective effect that alkalinity has on larger lake sturgeon. There would also be greater likelihood that older, larger YOY lake sturgeon may have already migrated to lower reaches of the stream or into the lakes where TFM concentrations would be less (Johnson *et al.*, 1999). By combining this strategy with other approaches, such as using TFM:niclosamide mixtures in sturgeon streams, lake sturgeon vulnerability to lampricide treatments could be lowered even further.

Finally, our findings demonstrate that sea lamprey control using TFM and lake sturgeon conservation are not incompatible. With a better understanding of how lampricides are handled and detoxified by sturgeon, along with improved knowledge of their life history, movements and behaviour within the Great Lakes tributaries, we believe it will be possible to adopt measures that could contribute to the twin goals of sea lamprey control and lake sturgeon conservation.
